# The Development of Object Function and Manipulation Knowledge: Evidence from a Semantic Priming Study

**DOI:** 10.3389/fpsyg.2016.01239

**Published:** 2016-08-23

**Authors:** Cynthia Collette, Isabelle Bonnotte, Charlotte Jacquemont, Solène Kalénine, Angela Bartolo

**Affiliations:** ^1^Univ. Lille, CNRS, CHU Lille, UMR 9193 - SCALab - Sciences Cognitives et Sciences AffectivesLille, France; ^2^Institut Universitaire de FranceParis, France

**Keywords:** manipulation knowledge, function knowledge, action semantics, developmental study, priming paradigm

## Abstract

Object semantics include object function and manipulation knowledge. Function knowledge refers to the goal attainable by using an object (e.g., the function of a key is to open or close a door) while manipulation knowledge refers to gestures one has to execute to use an object appropriately (e.g., a key is held between the thumb and the index, inserted into the door lock and then turned). To date, several studies have assessed function and manipulation knowledge in brain lesion patients as well as in healthy adult populations. In patients with left brain damage, a double dissociation between these two types of knowledge has been reported; on the other hand, behavioral studies in healthy adults show that function knowledge is processed faster than manipulation knowledge. Empirical evidence has shown that object interaction in children differs from that in adults, suggesting that the access to function and manipulation knowledge in children might also differ. To investigate the development of object function and manipulation knowledge, 51 typically developing 8-9-10 year-old children and 17 healthy young adults were tested on a naming task associated with a semantic priming paradigm (190-ms SOA; prime duration: 90 ms) in which a series of line drawings of manipulable objects were used. Target objects could be preceded by three priming contexts: related (e.g., knife-scissors for function; key-screwdriver for manipulation), unrelated but visually similar (e.g., glasses-scissors; baseball bat-screwdriver), and purely unrelated (e.g., die-scissors; tissue-screwdriver). Results showed a different developmental pattern of function and manipulation priming effects. Function priming effects were not present in children and emerged only in adults, with faster naming responses for targets preceded by objects sharing the same function. In contrast, manipulation priming effects were already present in 8-year-olds with faster naming responses for targets preceded by objects sharing the same manipulation and these decreased linearly between 8 and 10 years of age, 10-year-olds not differing from adults. Overall, results show that the access to object function and manipulation knowledge changes during development by favoring manipulation knowledge in childhood and function knowledge in adulthood.

## Introduction

The way humans interact with objects has been a matter of debate since the sensory-functional theory was proposed by Warrington and Shallice (1984) on the distinction between living (e.g., plants and animals) and non-living (i.e., manmade objects) conceptual categories. According to this theory, functional rather than visual features are relevant to distinguish non-living from living items. Therefore, according to the sensory-functional theory, objects (but not animals or vegetables) are categorized on the basis of their functional features. However, functional features are defined broadly in this framework and often include many subtypes of non-perceptual features, in particular manipulation features. Thus, the relative role of function and manipulation knowledge in object concepts is still poorly understood.

In patient and neuroimaging studies, the term “function knowledge” usually refers to the goal attainable by using an object (e.g., the function of a key is to open or close a door). The term “manipulation knowledge” refers to gestures one has to execute to use an object appropriately (e.g., a key is held between the thumb and the index fingers, inserted into the door lock and then turned) and implies motor-based simulation (Decety et al., 1994, 1997; Stephan et al., 1995; Bartolo et al., 2007). The first studies in patients drew attention to the role of manipulation knowledge in retrieving object concept. Sirigu et al. (1991) reported on a patient with visual agnosia, a condition characterized by a deficit in visual object recognition, who, despite his difficulties in recognizing objects presented visually, demonstrated preserved knowledge about the way some of the objects he could not recognize were manipulated. In another study, Magnié et al. (1998) described a similar case of a patient with semantic agnosia who could correctly manipulate some objects that he could not name. Interestingly, in some cases, the object name was appropriate with the gesture performed (e.g., he correctly showed the gesture associated with a key but called the object a screwdriver). The relationship between object function and manipulation knowledge was posited by Buxbaum et al. (2000). They reported two patients with left brain damage who had a preserved capacity to retrieve object function knowledge coupled with difficulties in recovering object manipulation knowledge. In a subsequent study, double dissociation between object function and manipulation knowledge was reported in left-brain damage patients with or without limb apraxia (Buxbaum and Saffran, 2002). Apraxic patients exhibited preserved object function knowledge and impaired manipulation knowledge, whereas patients without apraxia showed the opposite profile. Taken together, these results suggest that difficulties in accessing object function knowledge do not prevent patients from having preserved knowledge of the way in which the object is manipulated. On the other hand, they imply that object manipulation knowledge, but not object function knowledge, is necessary to interact correctly with objects. Therefore, patient studies indicate that knowledge about object function and manipulation should be considered separately.

**Figure 1:**
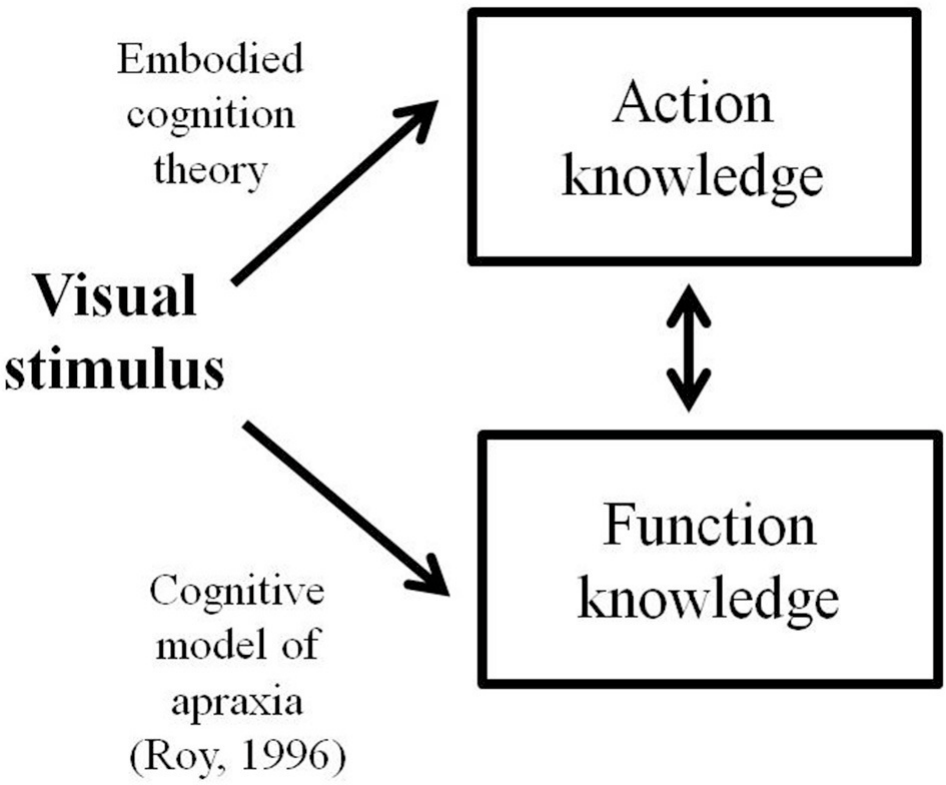
**Schematic representation of object processing according to the embodied cognition theory and the cognitive model of limb apraxia proposed by Roy (1996)**.

The distinction between object function and manipulation knowledge has been further investigated in neuroimaging and brain lesions analyses studies (Binkofski et al., 1999; Chao et al., 1999; Chao and Martin, 2000; Tranel et al., 2003; Boronat et al., 2005). In particular, in an fMRI block design experiment, Boronat et al. (2005) have failed to find substantial differences in the neural substrates of manipulation and function knowledge: both recruit similar fronto-parietal regions of the visuo-motor system, with greater activity during manipulation judgment. This result suggests that both function and manipulation knowledge rely on the same neural network and that manipulation knowledge requires additional sensory-motor components. This finding is consistent with embodied views of object concepts (Barsalou, 1999, 2008; Gallese and Lakoff, 2005) that consider that sensorimotor experience underlies conceptual retrieval, which, in the case of manipulable objects, would involve active and concurrent motor simulation through the activation of fronto-parietal circuits. In a subsequent fMRI study, dissociation between function and manipulation knowledge has been shown in an event-related design (Canessa et al., 2008). In particular, results showed activation of a left frontoparietal network comprising the intraparietal sulcus, the inferior parietal lobule and the dorsal premotor cortex for manipulation knowledge relative to function knowledge; and activation of the anterior inferotemporal cortex for function knowledge relative to manipulation knowledge. Such neuroanatomical dissociation between function and manipulation knowledge is in line with the distinction within the conceptual system proposed in the cognitive model of limb apraxia by Roy (1996; see also the updated version by Stamenova et al., 2012). To account for the capacity of patients to use objects (or executing a correct pantomime of visually presented objects), Roy conceived a conceptual system that contains “knowledge of tool/object function” (i.e., function knowledge) and “knowledge of action” (i.e., manipulation knowledge). In this model, the visual stimulus (i.e., the object) accesses object function knowledge before gaining access to manipulation knowledge. According to cognitive models of limb apraxia (Roy, 1996), visual objects first activate function knowledge before reaching manipulation knowledge. In contrast, the embodied cognition theorists postulate that motor simulation is a compulsory stage for retrieval of function features (Gallese and Lakoff, 2005). In other words, embodied cognition theorists consider that a visual stimulus accesses manipulation knowledge before function knowledge (see Figure 1). Taken as a whole, neuroanatomical findings are unable to differentiate two alternative predictions about the relative role of function and manipulation knowledge in object concepts.

Manipulation and function similarity judgments were assessed in a recent study in healthy adults (Garcea and Mahon, 2012). Participants had to choose between two objects the one that shared the same function or the same manner of manipulation as a target object. Results showed faster reaction times for objects related according to their function than for those related according to their manner of manipulation, leading the authors to affirm that manipulation knowledge is not necessary to retrieve function knowledge. In other terms, this suggests that access to function knowledge is faster than that to manipulation knowledge, which is exactly in agreement with the model proposed by Roy (1996). However, the use of explicit tasks in healthy participants, such as tasks based on similarity judgments, allows the relationship between function and manipulation knowledge to be tested but might not be adequate to investigate the way humans with normal brain functioning access these types of information. One relevant way to investigate access to semantic knowledge is to use implicit measures of semantic processing. Yet, to our knowledge, implicit processing of function and manipulation knowledge has never been directly compared.

Interesting findings have arisen from semantic priming and eye-tracking paradigms. On one hand, both object nouns and pictures implicitly activate manipulation knowledge (Myung et al., 2006; Labeye et al., 2008). In particular, Myung et al. (2006) first found faster lexical decisions on targets preceded by primes related in terms of manipulation (e.g., piano and typewriter) than by unrelated primes (e.g., blanket and typewriter). In a second experiment, participants were instructed to identify the picture that corresponded to a target object name in a visual display including distractor objects unrelated or related to the target in terms of manipulation. The monitoring of eye movements revealed that distractors related in terms of manipulation competed for attention more than unrelated objects during the word-to-picture matching task, indicating that manipulation knowledge participates in object identification. On the other hand, several studies in healthy adults have found that object functional attributes are also implicitly activated by visual objects by using similar priming and eye-tracking paradigms (Schreuder et al., 1984; Yee et al., 2011; Kalénine et al., 2012; Wamain et al., 2015). Thus, both function and manipulation knowledge may be implicitly activated during object visual processing. An important issue remaining is how these two types of knowledge are related in object concepts in adulthood and during development.

To our knowledge, no developmental study has yet evaluated manipulation and function knowledge together. Nevertheless, indirect arguments about the relative role of the two types of knowledge in object concepts may be found in the literature on thematic processing. Thematic relations concern objects that play a complementary role in a given scenario (Estes et al., 2011) and which can belong to the same superordinate category (e.g., hammer-nail) or not (e.g., spoon-yogurt). Using a priming paradigm, Perraudin and Mounoud (2009) studied the role of thematic (e.g., bottle—glass) and categorical (e.g., cup—glass) relations based on function similarity between objects in children of 5, 7, and 9 years of age as well as in a group of adults. Results showed that function priming effects appeared only at 7 years of age, and that 5-year-old children presented mainly thematic priming effects. They also observed that the size of thematic priming effects decreased as age increased, although it was still present in adults. The presence of a thematic priming effect in younger children, which was higher than in the other groups of participants, was related by the authors to the role of action in the development of object concepts. They proposed that at 5 years of age, access to the function of objects is strictly associated with the action that can be applied to the objects. In a previous study, Mounoud et al. (2007) explored the relationship between action and object representations by presenting a pantomime gesture as prime (e.g., hammering) followed by the associated object (i.e., hammer) in a naming task (experiment 1) and in a categorization task (experiment 2) in children from 5 to 12 years of age. In both experiments, action priming effects decreased as age increased, mirroring the developmental pattern observed for thematic priming effects. They assumed that objects are basically defined at first on the basis of the actions that can be accomplished with them. Therefore, they considered that the fact that similar actions can be attributed to different objects (e.g., the action of cutting can be attributed to a knife as well as to a hatchet) allows children to generate categories of objects (e.g., cutting objects, Lakoff, 1987). Subsequently, once the object semantic properties necessary to accomplish the action are extracted (e.g., cutting objects have a cutting edge), the role of actions decreases in the definition of concepts (see also Mandler, 1979; and Markman, 1981; Nelson, 1988).

Consistent results have been reported in recent eye-tracking studies contrasting thematic and function implicit processing in adults and in 6-, 8,- and 10-year-old children (Kalénine et al., 2012; Pluciennicka et al., 2016). Participants were asked to identify the picture that corresponded to a target object name in a visual display including distractor objects related or not to the target either thematically or in terms of function similarity. In adults, competition effects with thematically related distractors (e.g., saw and wood are used together) occurred earlier than the competition effect with functionally related distractors (saw and knife can both be used to cut things). Moreover, thematic competition effects were present from the age of 6 and remained stable until adulthood, whereas function competition effects showed a developmental trend and only emerged at 10 years of age. Taken together, developmental studies indicate that thematic knowledge develops earlier than function knowledge, and suggest that action representations underlie thematic processing of manipulable objects. Accordingly, manipulation knowledge may play a more important role in object identification than function knowledge in young children and this relative advantage may decrease with age until adulthood.

In the present study, we used a naming task associated with a semantic priming paradigm to investigate the role of function and manipulation knowledge in object identification in 8-, 9-, and 10-year-old children and in a group of adults. The priming context was manipulated by contrasting a related context (i.e., primes shared the same function, FR, or the same manipulation, MR, with the target object) to two unrelated contexts. In the “purely unrelated context” (UR), the prime and the target shared no semantic or visual relation, whereas in the “unrelated but visually similar” context (URVS), the prime and the target shared no semantic relation but were visually similar to the target. Specifically, we assessed the developmental trajectories of function and manipulation priming effects on naming. We predicted priming effects for objects related by their similar manipulation in the youngest children and expected this effect to decrease with age. On the other hand, function priming effects should emerge only later in development.

## Materials and methods

### Participants

Sixty-eight native French speakers with normal or corrected-to-normal vision participated in the experiment. All were right-handed (Edinburgh Inventory, Oldfield, 1971) and healthy. They were divided into four age groups of 17 participants each: 8-year-olds (3rd grade; *M* = 8.61 years [range: 8.25–8.91 years]; SD = 2.88 months; 6 females), 9-year-olds (4th grade; *M* = 9.83 years [9.33–10.17 years]; SD = 3.96 months; 11 females), 10-year-olds (5th grade; *M* = 10.79 years [10.17–11.33 years]; SD = 4.03 months; 11 females), and adults (*M* = 22.31 years [18–28 years]; SD = 20.8 months; 9 females). Children were recruited in three primary schools in a suburb of Lille, Hauts-de-France. Adults were undergraduate students of the University of Lille. All participants were naive to the purpose of the experiment. Participants (and their parents for minors) gave their written consent before beginning the experiment. The experimental protocol was approved by the local ethical committee in behavioral sciences of the University of Lille 3 (Ref. number 2013-4-S17) and conducted in accordance with the Declaration of Helsinki.

### Stimuli

The stimuli consisted in 128 line drawing pictures of manipulable objects (300 × 300 pixels). They were selected by means of a series of pretests conducted with no time pressure on a computer screen with young adults who were not included in the present experiment. The stimuli were kept only if they were correctly recognized (name agreement task) by a group of 20 participants. In addition, the similarity of prime-target pairs concerning their manipulation, their function, and their visual features was evaluated with three judgment tasks, each administrated to 10 participants. Responses were given on a digital keypad with a 7-point scale (1 = not at all similar, 7 = very similar). From the scores recorded in these three judgment tasks, we selected 32 targets (16 for the function condition and 16 for the manipulation condition), each associated with the three priming contexts, FR or MR, UR, URVS (see **Appendix**).

A series of *t*-tests was computed to statistically check the relevance of the selected prime-target pairs (Table 1). First, in the manipulation condition, the manipulation similarity score was higher for the MR (5.6) than for the UR (0.7) and URVS (0.6) contexts, *t*_(15)_ = 16.97, *p* < 0.001, Cohen's *d* = 4.24 and *t*_(15)_ = 18.39 *p* < 0.001, Cohen's *d* = 4.60, respectively. As expected, the visual similarity scores did not differ between the MR (3.2) and the URVS contexts (4.3), *t*_(15)_ = 2.18, *p* > 0.05, Cohen's *d* = 0.55, whereas they did between the MR (3.2) and the UR (0.5) contexts, *t*_(15)_ = 6.46, *p* < 0.001, Cohen's *d* = 1.61, and between the UR (0.5) and the URVS (4.3) contexts, *t*_(15)_ = 7.85 *p* < 0.001, Cohen's *d* = 1.96. Secondly, in the function condition, the function similarity score was higher for the FR context (5.8) than for the UR (0.1) and URVS (0.3) contexts, *t*_(15)_ = 28.26, *p* < 0.001, Cohen's *d* = 7.07, and *t*_(15)_ = 26.84, *p* < 0.001, Cohen's *d* = 6.71, respectively. Visual similarity scores did not differ between the FR (3.1) and the URVS (4.1) contexts, *t*_(15)_ = −1.53 *p* > 0.05, Cohen's *d* = 0.38, whereas they did between the FR (3.1) and the UR (0.3) contexts, *t*_(15)_ = 5.44, *p* < 0.001, Cohen's *d* = 1.36, and between the UR (0.3) and the URVS contexts (4.1), *t*_(15)_ = 10.45, *p* < 0.001, Cohen's *d* = 2.61. Furthermore, the manipulation similarity score was higher for the MR (5.6) than for the FR (1.9) contexts, *t*_(15)_ = 8.01, *p* < 0.001, Cohen's *d* = 2.0. Similarly, the function similarity score was higher for the FR (5.8) than for the MR (1.8) contexts, *t*_(15)_ = 10.11, *p* < 0.001, Cohen's *d* = 2.53.

**Table 1:** **Mean similarity scores and *standard deviations* in the judgment tasks**.

**Similarity Judgments**	**Manipulation**			**Function**		
	**MR**	**UR**	**URVS**	**FR**	**UR**	**URVS**
Manipulation	5.6	0.7	0.6	1.8	0.6	1.2
	0.6	0.7	0.9	1.4	0.7	1.3
Function	1.9	0.2	0.3	5.8	0.1	0.3
	2.0	0.4	0.6	0.6	0.3	0.6
Visual features	3.2	0.5	4.3	3.1	0.3	4.1
	1.4	0.8	1.6	1.7	0.6	1.5

*MR and FR: related; UR, purely unrelated; URVS, unrelated but visually similar*.

### Procedure

**Figure 2:**
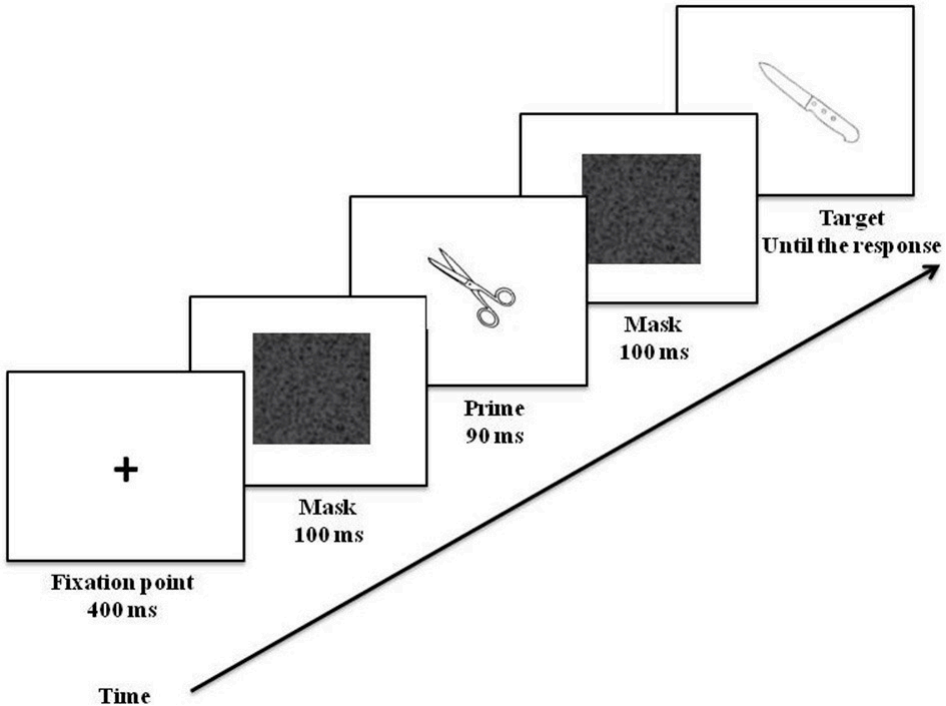
**Typical trial and timing of the experiment**. In the example, the target (knife) is preceded by a related prime (scissors) according to object function (i.e., cutting).

E-Prime 2.0 software (Psychology Software Tools, Pittsburgh, PA) was used to present the stimuli. It recorded reaction times and vocal responses as audio files via a microphone. The stimuli appeared on a white background on the center of a 15.4-inch screen with 60 Hz refresh rate. Three blocks of 32 prime-target pairs (16 for each condition) were created so that each target was associated with the three priming contexts, without any repetition of the target in a given block. Block order was counterbalanced across participants. Trial order within each block was randomized. On each trial, participants were first presented with a fixation cross for 400 ms, followed by a forward mask (a 300 × 300 pixel gray square similar to fuzzy television screen when signal is lost) lasting for 100 ms. Then the prime was presented for 90 ms, followed by a backward mask (similar to the forward mask) for 100 ms. In a previous EEG study it was shown that when a prime was presented for 90 ms, a semantic activation was observed (Eddy and Holcomb, 2010). At the offset of the backward mask, the target was displayed until the participants gave their response. Participants had to name the target as quickly and accurately as possible. The inter-trial interval (a blank screen) lasted 1000 ms. The experimental task was preceded by 32 practice trials. It took ~15 min to complete the task (Figure 2).

### Data analyses

Naming errors (including auto-corrections) were recorded in about 18% of the trials in the children groups and in 9% of trials in the adult group and were removed from RT analyses. For the priming analyses on correct RTs, first of all, RT smaller than 200 ms and larger than 10,000 ms were removed. Then, RTs above or lower than 3 standard deviations (SD) from the mean of each participant in each of the 6 conditions were excluded from the analyses. With this trimming method, 1.3% of the trials were excluded from the analyses. In the absence of *a priori* hypotheses, participant (*F*_1_) and item (*F*_2_) analyses on error percentages (i.e., naming errors and auto-corrections) were carried out by means of a three-way ANOVA, including the four groups (8-9-10 year-olds and adults) as a between-subject and within-item factor, condition (function and manipulation) as a within-subject and between-item factor, and priming context (related, purely unrelated, and unrelated but visually similar) as a within-subject and within-item factor.

The Shapiro–Wilk test and the Levene's test were used to verify distribution normality and variance homogeneity. Non-parametric alternative tests were used when assumptions were not respected. Specifically, children and adult performance was systematically contrasted using non-parametric tests to overcome variance heterogeneity between age groups. Priming effects were computed for each condition (manipulation and function) by subtracting the mean RTs in the related contexts (MR or FR) from the mean RTs in the UR context (see Table 2 for data on each condition by group). Therefore, positive values correspond to facilitation priming effects and negative values to interference priming effects.

**Table 2:** **Mean response times (ms), error percentages, and *standard deviations* as a function of group and condition**.

	**Manipulation**						**Function**					
	**MR**		**UR**		**URVS**		**FR**		**UR**		**URVS**	
	**RT**	**ER**	**RT**	**ER**	**RT**	**ER**	**RT**	**ER**	**RT**	**ER**	**RT**	**ER**
8-year-olds	1426	29.8	1668	25.4	1588	27.6	1341	18.4	1389	23.5	1320	23.5
	442	12.2	629	10.9	490	11.2	389	10.2	468	11.4	273	9.8
9-year-olds	1597	19.5	1539	19.1	1637	19.1	1499	12.9	1522	14.3	1457	12.9
	642	9.3	595	13.2	655	11.4	434	7.8	562	9.6	498	10.7
10-year-olds	1322	11	1199	14	1280	15.1	1234	12.5	1183	11.4	1212	12.5
	314	11.6	302	13.9	299	14.3	251	9.1	262	9.2	345	11.3
Adults	842	8.5	795	8.5	823	8.8	754	8.5	796	9.9	784	8.5
	148	5.8	98	7.3	115	6.7	82	9.1	82	9.4	101	10.1

*MR and FR: related; UR, purely unrelated; URVS, unrelated but visually similar, RT, Response time (ms); ER, Error rate*.

We hypothesized a differential developmental trajectory of manipulation and function priming effects. In young children, we expected facilitation priming effects in the manipulation condition but little impact of priming in the function condition. We further assumed that the facilitation priming effects of manipulation would decrease with age, whereas the development of function priming effects would show the opposite trajectory. To this aim and in the presence of *a priori* developmental hypotheses, we directly tested the evolution of priming effects between 8 and 10 years of age in each condition (manipulation and function) using linear and quadratic orthogonal contrasts. The linear contrast tested the difference between the youngest group (8-year-olds) and the oldest group of children (10-year-olds). The quadratic contrast compared the intermediate children group (9-year-olds) to the average of the two extreme children groups. A significant linear contrast associated with a non-significant quadratic contrast would reflect a linear development of priming effects between 8- and 10-year-olds (*cf*. Brauer and McClelland, 2005). Subsequently, we compared the priming effects exhibited by the oldest children (10-year-olds) and the group of adults in order to determine whether the former had reached an adult-like level of semantic processing.

Finally, we evaluated the contribution of visual similarity to the manipulation and function priming effects highlighted in the main analysis by considering the URVS context as baseline in priming effect computation (mean RTs URVS context—mean RTs MR or FR context).

## Results

### Errors

The three-way ANOVA on error percentages showed a main effect of group [*F*_1(3, 64)_ = 13.199, *p* < 0.001, $\eta_{p}^{2}$ = 0.38; *F*_2(3, 90)_ = 16.264, *p* < 0.001, $\eta_{p}^{2}$ = 0.25]. In both F1 and F2 analyses, Tukey's *post hoc* analyses showed that 8-year-olds committed more errors (24.7%) than any other group (9-year-olds: 16.3%, *p* < 0.01; 10-year-olds: 12.7%, *p* < 0.001; adults: 8.8%, *p* < 0.001) and that 9-year-olds also committed more errors than adults (*p* < 0.05). A main effect of condition in the by-subjects analysis was observed [*F*_1(1, 64)_ = 7.578, *p* < 0.008, $\eta_{p}^{2}$ = 0.11], with more errors in the manipulation condition (17.19%) than in the function condition (14.06%); however, this effect did not appear in the by-items analysis [*F*_2(1, 30)_ = 0.957, *p* = 0.336, $\eta_{p}^{2}$ = 0.03]. There was no main effect of priming context [*F*_1(2, 128)_ = 0.752, *p* = 0.473, $\eta_{p}^{2}$ = 0.01; *F*_2(2, 60)_ = 0.438, *p* = 0.647, $\eta_{p}^{2}$ = 0.01]. The interactions group × condition, group × priming context, and priming × condition were not significant [*F*_1(3, 64)_ = 1.963, *p* = 0.128, $\eta_{p}^{2}$ = 0.08 and *F*_2(3, 90)_ = 0.894, *p* = 0.45, $\eta_{p}^{2}$ = 0.03; *F*_1(6, 128)_ = 0.332, *p* = 0.91, $\eta_{p}^{2}$ = 0.02 and *F*_2(6, 180)_ = 0.263, *p* = 0.95, $\eta_{p}^{2}$ = 0.01; *F*_1(2, 128)_ = 0.915, *p* = 0.403, $\eta_{p}^{2}$ = 0.01 and *F*_2(2, 60)_ = 0.663, *p* = 0.52, $\eta_{p}^{2}$ = 0.02, respectively], nor was the group × condition × priming context interaction [*F*_1(6, 128)_ = 1.683, *p* = 0.13, $\eta_{p}^{2}$ = 0.07, and *F*_2(6, 180)_ = 1.662, *p* = 0.13, $\eta_{p}^{2}$ = 0.05].

### Manipulation priming effects (Figure 3)

#### Main analysis (UR—MR)

**Figure 3:**
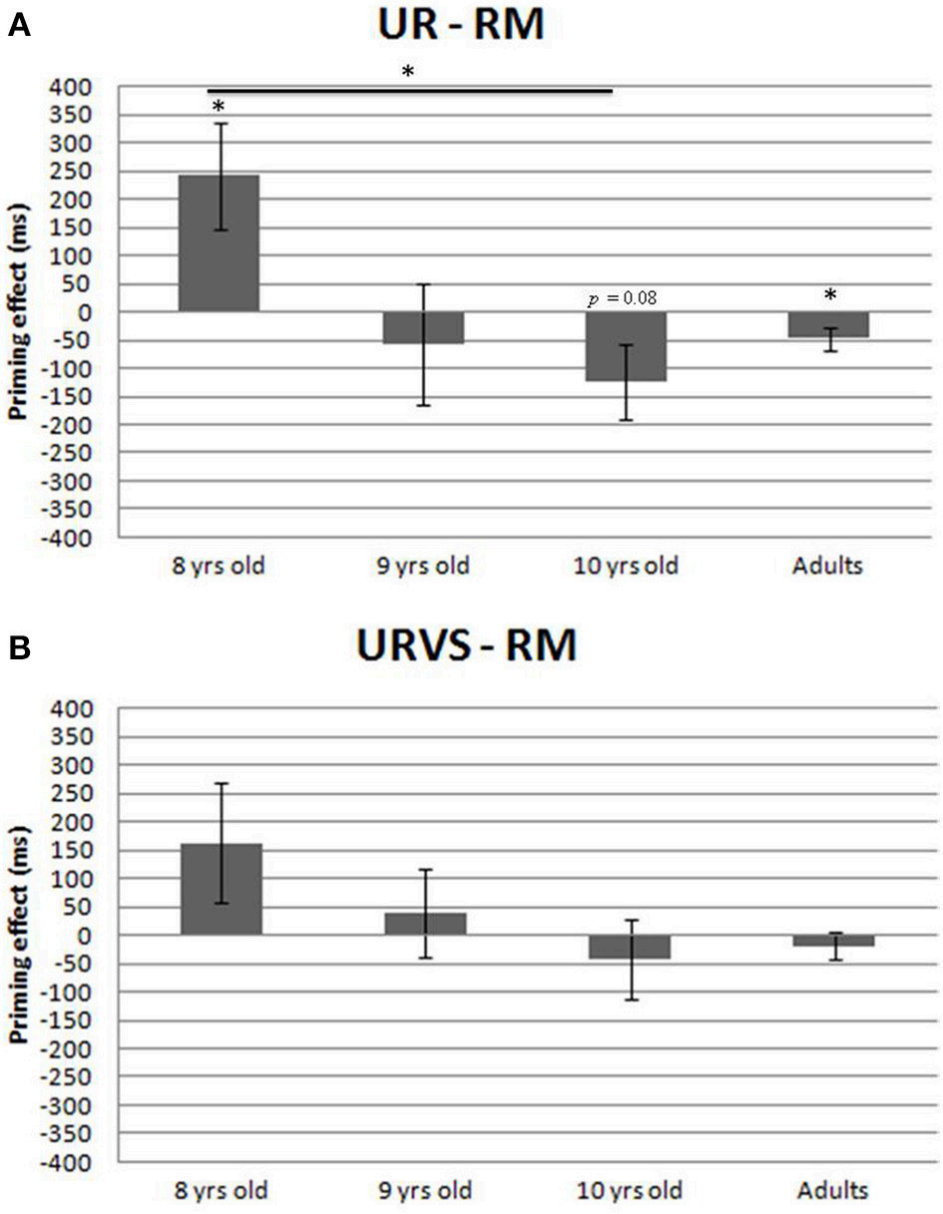
**(A)** Priming effects (UR—RM, in ms) and standard errors (by-subjects) registered in the manipulation condition in each group of participants. Asterisks indicate statistical differences (*p* < 0.05). **(B)** Priming effects (URVS—RM, in ms) and standard errors (by-subjects) registered in the manipulation condition in each group of participants.

The linear contrast between 8- and 10-year-olds was statistically significant [*F*_1(1, 48)_ = 8.13, *p* = 0.006, $\eta_{p}^{2}$ = 0.144; *F*_2(1, 15)_ = 10.75, *p* = 0.005, $\eta_{p}^{2}$ = 0.42], whereas the quadratic contrast (9-year-olds vs. extreme children groups) was not, [*F*_1(1, 48)_ = 1.11, *p* = 0.30, $\eta_{p}^{2}$ = 0.022; *F*_2(1, 15)_ = 1.74, *p* = 0.21, $\eta_{p}^{2}$ = 0.10], reflecting a linear decrease in manipulation priming effects between 8 and 10 years of age (Figure 3A). Moreover, the 10-year-olds did not differ from the adults [by-subjects: *Mann–Whitney U* = 97, *p* = 0.10, Cohen's *d* = 0.38; by-items, *Wilcoxon T* = 54, *p* = 0.47, Cohen's *d* = 0.44], indicating that manipulation priming effects had reached an adult-like level at 10 years of age. While manipulation priming facilitated object naming by 242 ms in the youngest group [by-subjects: *t*_(16)_ = 2.56, *p* = 0.02, Cohen's *d* = 0.62; by-items: *t*_(15)_ = 2.73, *p* = 0.015, Cohen's *d* = 0.68], it produced a cost of 47 ms in adults [by-subjects: *t*_(16)_ = −2.21, *p* = 0.042, Cohen's *d* = 0.53; by-items: *t*_(15)_ = −2.21, *p* = 0.043, Cohen's *d* = 0.55]. In the group of 10 year-olds, the cost of 124 ms reached a trend level of significance [by-subjects: *t*_(16)_ = −1.90, *p* = 0.08, Cohen's *d* = 0.46; by-items:, *t*_(15)_ = −1.95, *p* = 0.07, Cohen's *d* = 0.49].

#### Complementary analysis (URVS—MR)

When visual similarity was taken into account, manipulation priming was found neither to facilitate naming in 8-year-olds (163 ms) [by-subjects: *t*_(16)_ = 1.53, *p* = 0.14, Cohen's *d* = 0.37; by-items: *t*_(15)_ = 1.48, *p* = 0.16, Cohen's *d* = 0.37], nor to interfere with naming in adults (19 ms) [by-subjects: *t*_(16)_ = −0.78, *p* = 0.44, Cohen's *d* = 0.19; by-items: *t*_(15)_ = −1.04, *p* = 0.31, Cohen's *d* = 0.26], see Figure 3B.

### Function priming effects (Figure 4)

#### Main analysis (UR—FR)

**Figure 4:**
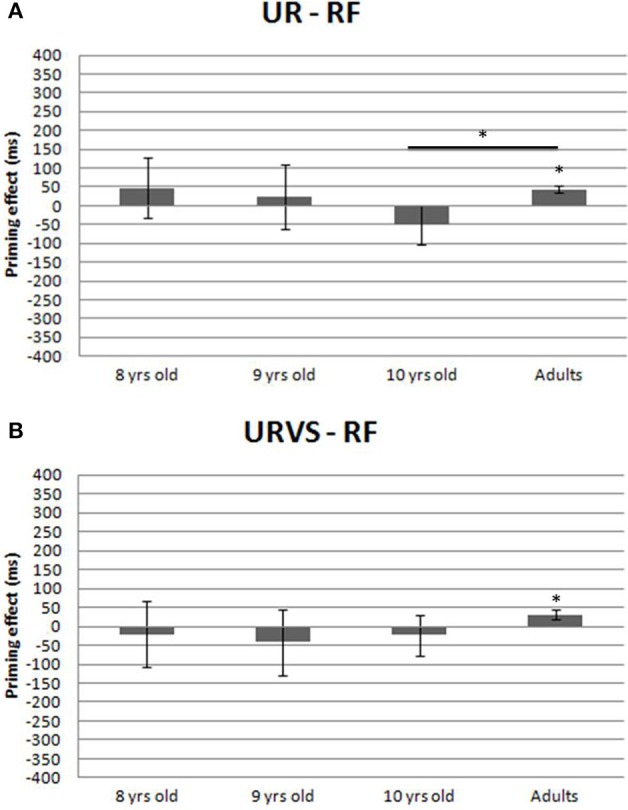
**(A)** Priming effects (UR—RF, in ms) and standard errors (by-subjects) registered in the function condition in each group of participants. Asterisks indicate statistical differences (*p* < 0.05). **(B)** Priming effects (URVS—RF, in ms) and standard errors (by-subjects) registered in the function condition in each group of participants. Asterisks indicate statistical differences (*p* < 0.05).

Neither the linear contrast between 8- and 10-year-olds [*F*_1(1, 48)_ = 0.88, *p* = 0.35, $\eta_{p}^{2}$ = 0.018; *F*_2(1, 15)_ = 1.01, *p* = 0.33, $\eta_{p}^{2}$ = 0.06] nor the quadratic contrast (9-year-olds vs. extreme children groups) [*F*_1(1, 48)_ = 0.077, *p* = 0.78, $\eta_{p}^{2}$ = 0.002; *F*_2(1, 15)_ = 0.007, *p* = 0.93, $\eta_{p}^{2}$ = 0.0005] were statistically significant. This highlights an absence of developmental change in function priming effects between 8 and 10 years of age (Figure 4A). Moreover, note that function priming effects were not significant in 8-year-olds (48 ms) [by-subjects: *t*_(16)_ = 0.58, *p* = 0.57, Cohen's *d* = 0.14; by-items: *t*_(15)_ = 0.69, *p* = 0.50, Cohen's *d* = 0.17] and in 10-year-olds (−51 ms) [by-subjects: *t*_(16)_ = −0.97, *p* = 0.34, Cohen's *d* = 0.33; by-items: *t*_(15)_ = −0.55, *p* = 0.59, Cohen's *d* = 0.14]. These emerged only in adults and facilitated naming by 43 ms [by-subjects: *t*_(16)_ = 4.81, *p* = 0.0002, Cohen's *d* = 1.16; by-items: *t*_(15)_ = 2.78, *p* = 0.013, Cohen's *d* = 0.70]. This priming effect was statistically different from that observed in 10-year-olds (−51 ms) by-subjects, but not by-items [by-subjects: *U* = 56, *p* = 0.02, Cohen's *d* = 0.61; by-items: *T* = 38, *p* = 0.12, Cohen's *d* = 0.44].

#### Complementary analysis (URVS—FR)

When visual similarity was taken into account, function priming still facilitated naming in adults (31 ms) [by subjects: *t*_(16)_ = 2.035, *p* = 0.03, Cohen's *d* = 0.57; by-items: *t*_(15)_ = 2.1, *p* = 0.05, Cohen's *d* = 0.51], see Figure 4B.

## Discussion

The aim of the present study was to test the access to function and manipulation knowledge in human development. To this end, a naming task associated with a semantic priming paradigm was used in three groups of children of 8, 9, and 10 years of age, as well as in a group of adults. The primes were line drawing pictures of objects that could be related to the target objects according to their function (FR) or manipulation (MR). Two unrelated contexts were included: in one case, the prime was purely unrelated (i.e., semantically and visually different from the target, UR), while in the other, it was semantically unrelated but visually similar to the target (URVS).

In patients and neuroimaging studies, a double dissociation between function and manipulation knowledge has been posited, suggesting the independence between these two types of knowledge. Although the comparison between manipulation and function knowledge has not been specifically addressed in development, results from studies investigating processing of thematic relations between objects suggest that action representations are active early in human development at around 5–6 years of age. In contrast, processing of function similarities between objects develops later at around 7–8 years of age (Perraudin and Mounoud, 2009; Pluciennicka et al., 2016).

In our study, the term “manipulation knowledge” refers to the gestures required to use an object appropriately. Therefore, it implies an access to action representations when processing objects related by their manner of manipulation. By investigating implicit access to function and manipulation knowledge, we expected to find priming effects for manipulation knowledge in the youngest children, whereas function knowledge priming was expected to emerge only later in human development. Our results confirm a developmental trend in the access to manipulation knowledge, in which a 242 ms facilitation priming effect was visible in younger children and vanished gradually with age. The lack of a difference between the oldest group of children (10 years old) and the adult group suggests that at 10 years of age the priming effect was indistinguishable from that in the group of adults, in the knowledge that in this group, naming targets preceded by objects related according to their manner of manipulation produced a cost of 47 ms. In accordance with previous developmental findings (Mounoud et al., 2007), we found a decrease in action priming with age. Yet instead of having no effect on naming in adulthood, object primes sharing manipulation features with the target interfered with naming in adults, a phenomenon that is likely to start from the age of 10. This indicates that from 10 years of age, manipulation knowledge is no longer central to object identity and suggests it becomes replaced by other orthogonal types of knowledge. This issue is developed below.

With respect to function knowledge, the opposite developmental profile was found. No developmental trend was found in the group of children and a facilitation effect (43 ms) was demonstrated only in the group of adults.

Previous studies showed that object function is processed at around 7–8 years of age, whereas action representations (i.e., processing of tool-recipient thematic relations) are active at 5–6 years of age. With our paradigm, we found that manipulation and function knowledge had an opposite impact on object-naming during development. Manipulation knowledge was processed at 8 years of age but its impact decreased with age, whereas processing of function knowledge emerged only later, as it appeared solely in the adult group.

### The role of visual similarity

Some authors have highlighted the role of visual features in constructing superordinate semantic categories (Quinn and Eimas, 1996; Sloutsky and Fisher, 2004). The prime-target pairs related according to their function and to their manner of manipulation included in our study could share some visual features. Therefore, we checked whether visual similarity could have played a role in the priming effects recorded. If this were to be the case, priming effects should disappear when visual similarity is held constant, i.e., when priming effects are computed in relation to the unrelated but visually similar priming context baseline (URVS). When controlling for visual similarity, both facilitation manipulation priming effects observed in 8-year-olds and inhibition manipulation priming effects observed in adults disappeared. Only function priming effects reported in adults remained visible. This supports the idea of an important role of visual features in object conceptual processing, not only in the first years of life but also in older children. Moreover, it suggests that processing of manipulation and visual features overlaps at least partially, while processing of function features may be more independent from visual features. This may explain why function priming effects emerge only after 10 years of age. The selective role of visual features in access manipulation knowledge may also account for the changes in the direction of the manipulation priming effects: 8-year-olds may place greater emphasis on visual features when processing objects than adults. With development, the reliance on other object features such as function may decrease the relevance of visual features. As a consequence, visual/manipulation priming would slow down object concept processing in adults.

The next section extends the discussion on the interplay between manipulation and function knowledge.

### Interplay in function and manipulation knowledge

In adults, the information driven by objects related according to their manner of manipulation produced a cost in object naming. On the other hand, a priming effect in function knowledge emerged in this group. Adults indeed have a preference for function knowledge and priming effects even differed from those in the oldest group of children. Roy (1996) suggested that visually presented objects are processed first according to the function they serve. Therefore, our findings in adults are in line with this view, and they are also in agreement with the results obtained in another study when explicit tasks were used (Garcea and Mahon, 2012).

However, this view does not fit with the findings in our groups of children. While facilitation effects were recorded in the youngest group of children in the manipulation knowledge condition, they disappeared with age. On the other hand, children did not process function knowledge, which was active only in adults. To explain this pattern of results, a more embodied view should be considered for this group. The embodied cognition theory highlights the role of active and concurrent sensorimotor simulation in object processing (Gallese and Lakoff, 2005). Results obtained in our groups of children are in line with this hypothesis; indeed, the priming effects recorded in children for pairs of items related to their manner of manipulation are in agreement with the idea that children rely on action representations to process object identification. The role of manipulation knowledge seems to be effective until 10 years of age, as the priming effect recorded in this group did not differ from that of the adults. Note, however, that the difference in variability between children and adult groups may have hidden potential differences in manipulation priming amplitude between 10 year-olds and adults. Future research may want to investigate the development of manipulation and function priming in a broader life span perspective in order to better understand how priming effects stabilize after 10 years of age.

Taken together these findings suggest that early in development children benefit from information about the way objects are manipulated thanks to visual features, whereas little emphasis is placed on their function.

Our results suggest a shift in the use of the two types of semantic knowledge in the course of development. One possible explanation involves the type of interaction children establish with objects. Indeed, object manipulation is gradually acquired in human development. In human development, infants interact with objects before knowing what an object is for (von Hofsten, 2007). Pediatric studies have shown that the capacity to perform a correct reach-to-grasp action toward an object is attained at 13 months, an age at which infants are able to adjust their hand to the size of the object during the approach (von Hofsten, 2007). The ability to accomplish simple manipulable gestures is achieved at around 26 months, an age at which infants show the ability to rotate an object before inserting it into a hole (Örnkloo and von Hofsten, 2007). When exposed to a novel tool, infants learn about which part of the tool is meant to be held (Barrett et al., 2007), suggesting that prior experience with tools is important to understanding how the object is used rather than learning about its function. However, the capacity to manipulate objects similar to that of adults is acquired later in development (von Hofsten and Rönnqvist, 1988; Choudhury et al., 2007). Our results show that once object function knowledge is acquired, subsequent interactions with objects focus on knowledge based on *what an object is for* rather than on *how the object is used*. Children can use a hammer by hammering on any surface (manipulation knowledge being active), whereas adults focus on the functional meaning of the action that can be performed with it (i.e., the object is to hammer in a nail, i.e., function knowledge).

### Neuroanatomical development of structures subtending function and manipulation knowledge

Previous neuroimaging studies have shown neuroanatomical dissociation between function and manipulation knowledge. The left frontoparietal regions including the intraparietal sulcus, the inferior parietal lobule and the dorsal premotor cortex are specifically activated for manipulation knowledge; the anterior inferotemporal cortex is activated for function knowledge (Canessa et al., 2008). Our results show that manipulation knowledge is active early in development but that its role diminishes with age to a point at which a switch to function knowledge occurs. To account for this switch, a glance to human cortical development can be useful. Human cortical developmental studies have shown that the maturation of gray matter starts in the parietal lobe, spreading over the frontal, the occipital, and only finally over the temporal cortex (Gogtay et al., 2004). Maturation of the parietal lobe reaches its maximal volume in gray matter at 10–12 years of age (Giedd et al., 1999; Lenroot and Giedd, 2006), whereas the temporal lobe cortical gray matter peaks at around 16 years (Giedd et al., 1999).

Taken together, neuroanatomical changes in human development can be related to the type of cognitive functions that prevail in childhood. In the present study, we tested children from 8 to 10 years of age. At this age, the parietal lobe subserving manipulation knowledge is developing and the children could have called upon such knowledge. On the other hand, the temporal regions develop later at around 16 years of age. Since the temporal lobe could play a specific role in processing object concepts, it might take over the role previously played by the parietal lobe once it has developed.

In conclusion, this study showed for the first time that relative access to object function and manipulation knowledge changes during human development: manipulation knowledge is prominent in childhood whereas function knowledge is in adulthood.

## Author contributions

AB, IB, SK, CC, and CJ collaborated in specifying the experimental design. CC and CJ collected data. AB, IB, and SK participated in data analyses. AB, IB, SK, CC, and CJ contributed to the writing of the article.

### Conflict of interest statement

The authors declare that the research was conducted in the absence of any commercial or financial relationships that could be construed as a potential conflict of interest.

## Appendix

**Table A1:** **Stimuli used in the experiment**.

**Targets**		**Primes**					
**FUNCTION**		**FR**		**UR**		**URVS**	
Accordion	*Accordéon*	Violin	*Violon*	Tube	*Tube*	Schoolbag	*Cartable*
Aquarium	*Aquarium*	Bird shelter	*Abri à oiseau*	Wheelbarrow	*Brouette*	Soccer ball	*Ballon de foot*
Candle	*Bougie*	Lamp	*Lampe*	Belt	*Ceinture*	Spear	*Lance*
Lighter	*Briquet*	Matchbox	*Boîte d'allumettes*	Christmas ball	*Boule de noël*	Glue stick	*Baton de colle*
Fishing rod	*Canne à pêche*	Fishnet	*Filet de pêche*	Toilet brush	*Brosse wc*	Flute	*Flûte*
Scissors	*Ciseaux*	Knife	*Couteau*	Dice	*Dé*	Glasses	*Lunettes*
Eraser	*Gomme*	Chalk eraser	*Brosse de tableau*	Umbrella	*Parapluie*	Bandage	*Pansement*
Magnifying glass	*Loupe*	Bioculars	*Jumelles*	Cushion	*Coussin*	Medal	*Médaille*
Puzzle piece	*Pièce de puzzle*	Domino	*Domino*	Cotton bud	*Coton-tige*	T-shirt	*Débardeur*
Radio	*Radio*	Mp3 player	*Baladeur mp3*	Witch hat	*Chapeau de sorcière*	Sponge	*Éponge*
Dust collector	*Ramasse-poussières*	Vacuum cleaner	*Aspirateur*	Battery	*Pile*	Cap	*Casquette*
Backpack	*Sac à dos*	Suitcase	*Valise*	Needle	*Aiguille*	Boxing glove	*Gant de boxe*
Saw	*Scie*	Axe	*Hache*	Bowl	*Bol*	Comb	*Peigne*
Cellphone	*Téléphone portable*	Bottle at sea	*Bouteille à la mer*	Plate	*Assiette*	Deckchair	*Transat*
Fan	*Ventilateur*	Ventilator	*Éventail*	Remote control	*Télécommande*	Bicycle wheel	*Roue de vélo*
Steering wheel	*Volant de voiture*	Bicycle handlebar	*Guidon de vélo*	Notebook	*Cahier*	Clock	*Horloge*
**MANIPULATION**		**MR**		**UR**		**URVS**	
Watering can	*Arrosoir*	Teapot	*Théière*	Chain	*Chaine*	Bow	*Arc*
Writing pad	*Bloc-notes*	Yoghurt cup	*Pot de yaourt*	Sword	*Épée*	Card	*Carte*
Shopping trolley	*Chariot de courses*	Mower	*Tondeuse à gazon*	Fabric roll	*Rouleau de tissu*	Heater	Radiateur
Nail clippers	*Coupe-ongles*	Perfume	*Parfum*	Magic wand	*Baguette magique*	Playground slide	*Toboggan*
Iron	*Fer à repasser*	Brush	*Brosse*	Bird cage	*Cage à oiseau*	Small boat	*Barque*
Toaster	*Grille-pain*	Shutter	*Volet*	Guitar	*Guitare*	Cardboard box	*Carton*
Lantern	*Lanterne*	Handbag	*Sac à main*	Ruler	*Règle*	Scale	*Balance*
Measuring tape	*Mètre*	Zipper	*Braguette*	Boot	*Botte*	Skipping rope	*Corde à sauter*
Laundry basket	*Panier à linge*	Tray	*Plateau*	Case	*Trousse*	Top hat	*Chapeau*
Piano	*Piano*	Computer	*Ordinateur*	Trousers	*Pantalon*	Pizza box	*Boite à pizza*
Pipe	*Pipe*	Whistle	*Sifflet*	Stool	*Tabouret*	Ladle	*Louche*
Water gun	*Pistolet à eau*	Spray	*Vaporisateur*	Necklace	*Collier*	Sewing machine	*Machine à coudre*
Rake	*Rβteau*	Squeegee	*Balai-raclette*	Ring	*Bague*	Kid scooter	*Trottinette*
Tap	*Robinet*	Jam pot	*Pot de confiture*	Electric plug	*Prise électrique*	Heel shoe	*Escarpin*
Drum	*Tambour*	Hammer	*Marteau*	Stamp	*Timbre*	Button	*Bouton*
screwdriver	tournevis	key	clef	tissue	mouchoir	baseball bat	batte de baseball
